# Synthetic Prostacyclin Agonist Attenuates Pressure-Overloaded Cardiac Fibrosis by Inhibiting FMT

**DOI:** 10.1016/j.omtm.2020.09.005

**Published:** 2020-09-16

**Authors:** Kenta Masada, Shigeru Miyagawa, Yoshiki Sakai, Akima Harada, Tomomitsu Kanaya, Yoshiki Sawa

**Affiliations:** 1 Department of Cardiovascular Surgery, Osaka University Graduate School of Medicine, Osaka 565-0871, Japan

**Keywords:** ONO-1301, prostacyclin, cardiac fibrosis, fibroblast

## Abstract

Fibroblast-to-myofibroblast transition (FMT) is the primary inducer of cardiac fibrosis. ONO-1301, a synthetic prostacyclin agonist, reportedly promotes tissue fibrosis repair by enhancing anti-fibrotic cytokine production. We hypothesized that ONO-1301 attenuates pressure-overloaded cardiac fibrosis by modulating FMT and generated a pressure-overloaded murine model via transverse aortic constriction (TAC) to evaluate the *in vivo* effects of ONO-1301. Cardiac fibrosis, left ventricular dilatation, and systolic dysfunction were established 4 weeks after TAC; however, ONO-1301 treatment initiated 2 weeks after TAC significantly attenuated those effects. Furthermore, ONO-1301 treatment significantly upregulated expression levels of cardioprotective cytokines such as vascular endothelial growth factor and hepatocyte growth factor in TAC hearts, whereas FMT-related factors, including transforming growth factor (TGF)-β1 and connective tissue growth factor, were significantly downregulated. The number of α-smooth muscle actin (α-SMA)- and vimentin-positive cells, representing fibroblast-originated cells transitioned into myofibroblasts, was significantly reduced in ONO-1301-treated TAC hearts. We isolated cardiac fibroblasts (CFs) from the left ventricles of adult male mice and assessed the effects of ONO-1301 on CFs stimulated by TGF-β. Results showed that ONO-1301 co-incubation significantly suppressed TGF-β-induced α-SMA expression and collagen synthesis, and significantly inhibited TGF-β-induced CF proliferation and migration. Our findings suggest that ONO-1301 ameliorates pressure overloaded cardiac fibrosis by inhibiting TGF-β-induced FMT.

## Graphical Abstract

**Figure:**
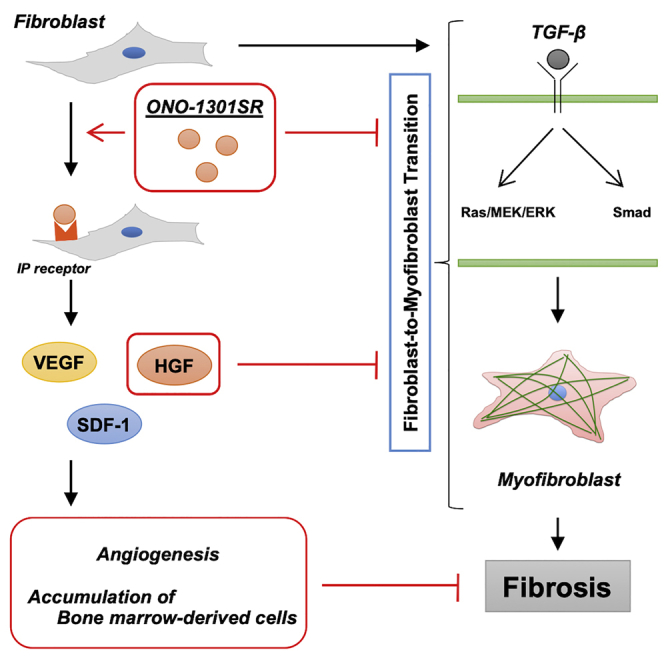

## Teaser

ONO-1301SR, a sustained-release drug-delivery system of a synthetic prostacyclin agonist, attenuates pressure overload-induced cardiac fibrosis by inhibiting TGF-β-induced fibroblast-to-myofibroblast transition in addition to enhancing the production of protective cytokines, such as hepatic growth factor (HGF), vascular endothelial growth factor (VEGF), and stromal cell-derived factor 1 (SDF-1).

## Introduction

Cardiac fibrosis is a feature of myocardial failure and is associated with increased ventricular stiffness, arrhythmia, diastolic dysfunction, and combined systolic and diastolic dysfunction.^1^ The severity of cardiac fibrosis has been reported to be a predictor of mortality in patients with heart failure caused by hypertension, valvular heart disease, and nonischemic dilated cardiomyopathy.^2^^,^^3^ Thus, cardiac fibrosis is considered a key target in the treatment of heart failure. However, effective anti-fibrotic therapies have not been established.^4^

Cardiac fibrosis is characterized by excessive deposition of extracellular matrix (ECM) proteins by cardiac fibroblasts (CFs), which reduces tissue compliance and leads to the progression of heart failure.^5^ After a myocardial injury, CFs are activated and transformed to the myofibroblast phenotype and secrete elevated levels of collagen and other ECM proteins. Transforming growth factor β (TGF-β) signaling plays a pivotal role in the process of accumulation of CFs and fibroblast-to-myofibroblast transition (FMT).^6^ Previous studies using animal models suggested that cardiac fibrosis could be prevented by the inhibition of TGF-β. However, TGF-β-based anti-fibrotic therapies have been limited due to their adverse effects on immune function.^7^^,^^8^

ONO-1301, a synthetic prostacyclin agonist, has long-lasting prostacyclin activity and thromboxane synthase inhibitory activity, and it is chemically and biologically stable, as it lacks the typical prostanoid structures; therefore, ONO-1301 has several theoretical advantages as a therapeutic agent over other synthetic prostacyclin agonists.^9^ Furthermore, the poly-lactic *co*-glycolic acid (PLGA)-polymerized form of ONO-1301, ONO-1301SR, was developed to achieve a further slow-releasing system of this agent into targeted tissue, and our laboratory and others have reported the beneficial effects of ONO-1301SR in various animal models of heart failure.^10^, ^11^, ^12^, ^13^, ^14^, ^15^ Although there are some agents that are similar to ONO-1301, a sustained release form of a prostacyclin agonist has been developed only for ONO-1301.

ONO-1301SR has a high binding affinity for prostaglandin I_2_ receptors (IPs) on endothelial cells, vascular smooth muscle cells, or fibroblasts, and stimulation of IPs promotes cAMP (cyclic adenosine monophosphate) elevation, leading to upregulation of expression of various protective cytokines such as hepatic growth factor (HGF), vascular endothelial growth factor (VEGF), and stromal cell-derived factor 1 (SDF-1), contributing to the repair of cardiac fibrosis.^10^, ^11^, ^12^, ^13^, ^14^, ^15^ In addition, ONO-1301SR has also been reported to ameliorate renal interstitial fibrosis by inhibiting TGF-β-induced endothelial-mesenchymal transition.^16^ Furthermore, other synthetic prostacyclin agonists, beraprost and iloprost, have been reported to inhibit TGF-β-induced CF activation.^17^^,^^18^ Taken together, ONO-1301SR is expected to play a role as a new anti-fibrotic drug for cardiac fibrosis by modulating TGF-β-induced FMT in addition to enhancing the production of protective cytokines; however, there is no report on the anti-fibrotic effects of ONO-1301SR from the viewpoint of modulating FMT. Therefore, in this study, we hypothesized that ONO-1301SR attenuates pressure-overloaded cardiac fibrosis by inhibiting FMT in a mouse transverse aortic constriction (TAC) model.

## Results

### ONO-1301SR Prevents Cardiac Hypertrophy and Dysfunction Induced by TAC

**Figure 1:**
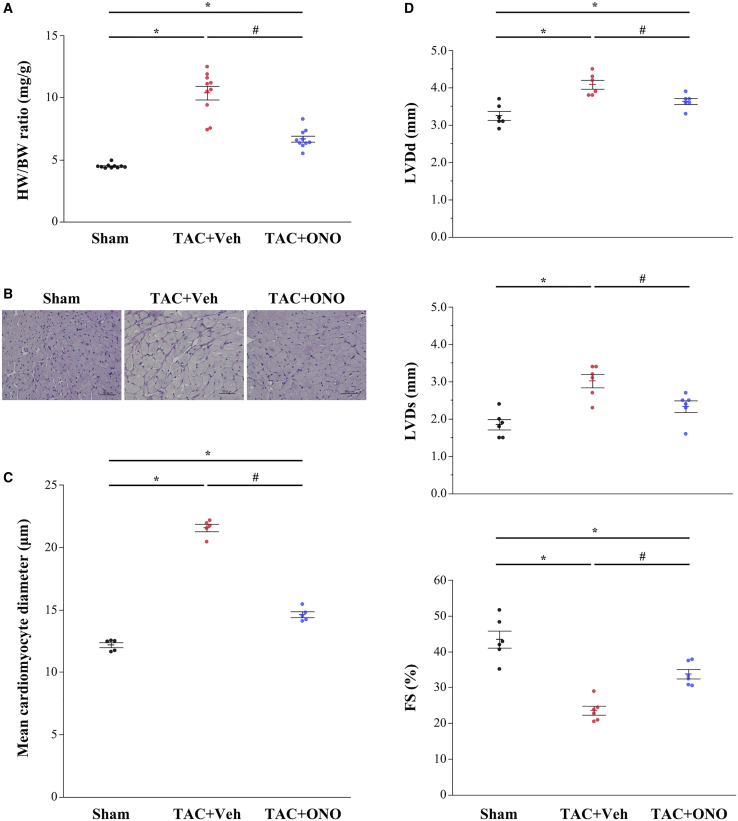
ONO-1301SR Prevents Pressure Overload-Induced Cardiac Hypertrophy and Systolic Dysfunction (A) Heart weight (HW) to body weight (BW) ratio at 4 weeks after sham or transverase aortic constriction (TAC) surgery (n = 10 for each group). (B) Histological manifestation of cardiomyocytes with periodic acid-Schiff (PAS) staining. Original magnification, ×40. (C) Mean cross-sectional diameter of cardiomyocytes (n = 5 for each group). (D) Echocardiographic parameters (n = 6 for each group). Data are represented as mean ± SEM. ∗p < 0.05 versus the sham group; ^#^p < 0.05 versus the vehicle group. TAC+Veh, TAC-operated mice treated with vehicle (the vehicle group); TAC+ONO, TAC-operated mice treated with ONO-1301SR (the ONO group); LVDd, left ventricular end-diastolic diameter; LVDs, left ventricular end-systolic diameter; FS, fractional shortening; LV, left ventricular.

The effect of ONO-1301SR on TAC-induced cardiac hypertrophy is shown in Figure 1A. The heart weight (HW) to body weight (BW) ratio significantly increased in the vehicle group compared to that in the sham group (10.4 ± 0.6 versus 4.5 ± 0.1, p < 0.01), but it was significantly reduced in the ONO group relative to that in the vehicle group (6.7 ± 0.2, p < 0.01). As shown in Figures 1B and 1C, histological examination demonstrated a significantly smaller cross-sectional diameter of cardiomyocytes in the ONO than in the vehicle group (14.6 ± 0.2 μm versus 21.6 ± 0.7 μm, p = 0.012). Additionally, the left ventricular (LV) end-diastolic diameter (LVDd, mm) and end-systolic diameter (LVDs, mm) were significantly increased, and systolic cardiac systolic function was significantly suppressed in the vehicle group compared with that in the sham group (Figure 1D); ONO-1301SR administration significantly prevented TAC-induced LV dilatation and systolic dysfunction.

### ONO-1301SR Attenuates TAC-Induced Cardiac Fibrosis and Collagen Expression

**Figure 2:**
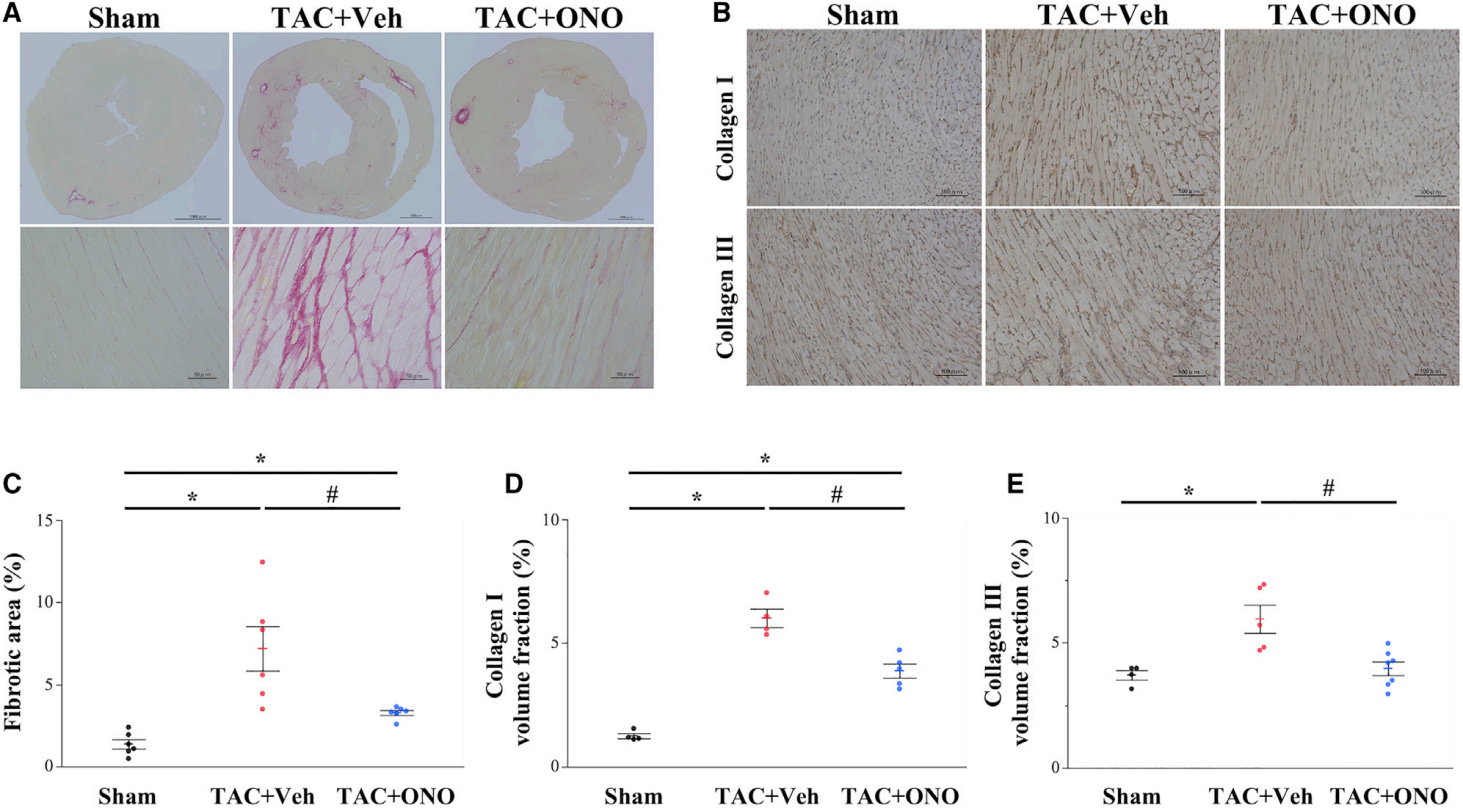
ONO-1301SR Attenuates Pressure Overload-Induced Cardiac Fibrosis and Collagen Expression (A) Histological manifestation of the left ventricular sections with picrosirius red staining. Original magnification, ×40 (top), ×400 (bottom). (B) Immunohistochemistry of type I and type III collagen. Original magnification, ×200. (C) Quantification of the fibrotic area (n = 6 for each group). (D and E) Quantification of collagen volume fraction (n = 5‒8 for each group) (D, type I collagen; E, type III collagen). Data are represented as mean ± SEM. ∗p < 0.05 versus the sham group; ^#^p < 0.05 versus the vehicle group. TAC+Veh, TAC-operated mice treated with vehicle (the vehicle group); TAC+ONO, TAC-operated mice treated with ONO-1301SR (the ONO group).

**Figure 3:**
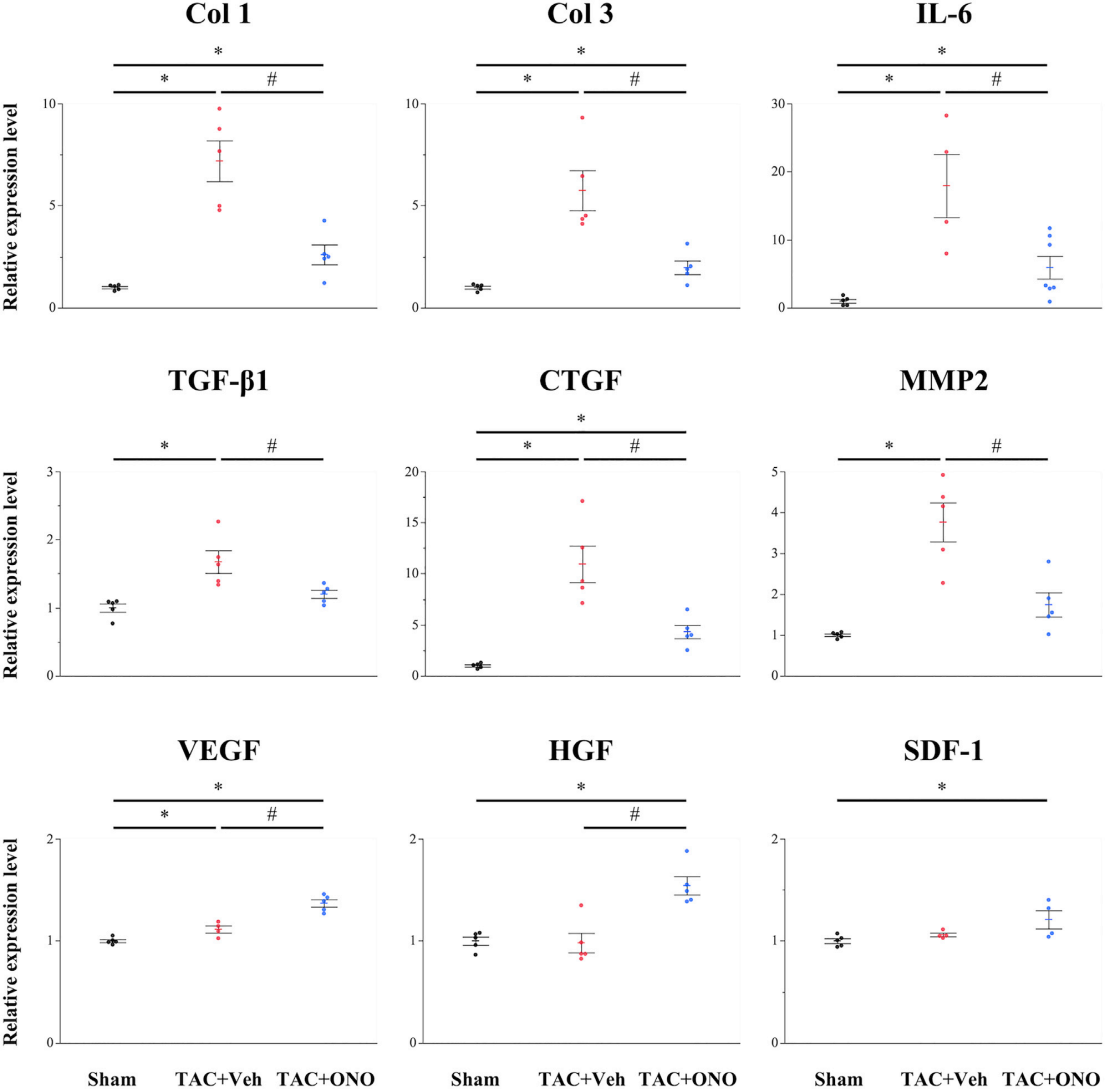
Gene Expression in the Murine Left Ventricles Gene expression of type I collagen (*Col1*), *Col3*, interleukin (*IL*)*-6*, transforming growth factor (*TGF*)*-β1*, connective tissue growth factor (*CTGF*), and matrix metalloproteinase 2 (*MMP2*) was significantly lower in the ONO group than in the vehicle group, whereas gene expression of vascular endothelial growth factor (*VEGF*), hepatocyte growth factor (*HGF*), and stromal cell-derived factor (*SDF*)*-1* was significantly higher in the ONO group (n = 5‒8 for each group). Data are represented as mean ± SEM. ∗p < 0.05 versus the sham group; ^#^p < 0.05 versus the vehicle group.

Picrosirius red staining (Figure 2A) showed that cardiac fibrosis, especially interstitial fibrosis, was significantly increased in the vehicle compared to that in the sham group, whereas ONO-1301SR treatment significantly attenuated TAC-induced cardiac fibrosis. Furthermore, the fibrotic area was significantly lower in the ONO than that in the vehicle group (Figure 2C; 3.3% ± 0.2% versus 7.2% ± 1.4%, p = 0.010). Immunohistochemical staining with type I collagen (Col1) and Col3 (Figure 2B) revealed collagen volume fractions to be significantly lower in the ONO than in the vehicle group (Figures 2D and 2E). Furthermore, gene expression levels of *Col1* and *Col3* in murine hearts were significantly downregulated in the ONO group than those in the vehicle group (Figure 3).

### Effect of ONO-1301SR on Gene Expression in the TAC Heart

Gene expression of various cytokines in the left ventricles was assessed using quantitative real-time polymerase chain reaction (PCR). As shown in Figure 3, fibrosis-related cytokines, such as TGF-β1 and connective tissue growth factor (CTGF), which is reported to be a central mediator of tissue fibrosis,^19^ were significantly upregulated by TAC, but significantly suppressed by ONO-1301SR treatment. Matrix metalloproteinase 2 (MMP2), the marker responsible for maintaining ECM integrity, and interleukin (IL)-6, one of the representative inflammatory cytokines,^20^ were also significantly upregulated in the vehicle group, but significantly suppressed in the ONO group. Conversely, protective cytokines, such as HGF, VEGF, and SDF-1, were significantly upregulated in the ONO group compared to levels in the sham or vehicle groups.

### ONO-1301SR Suppresses the Number of Myofibroblasts in the TAC Heart

**Figure 4:**
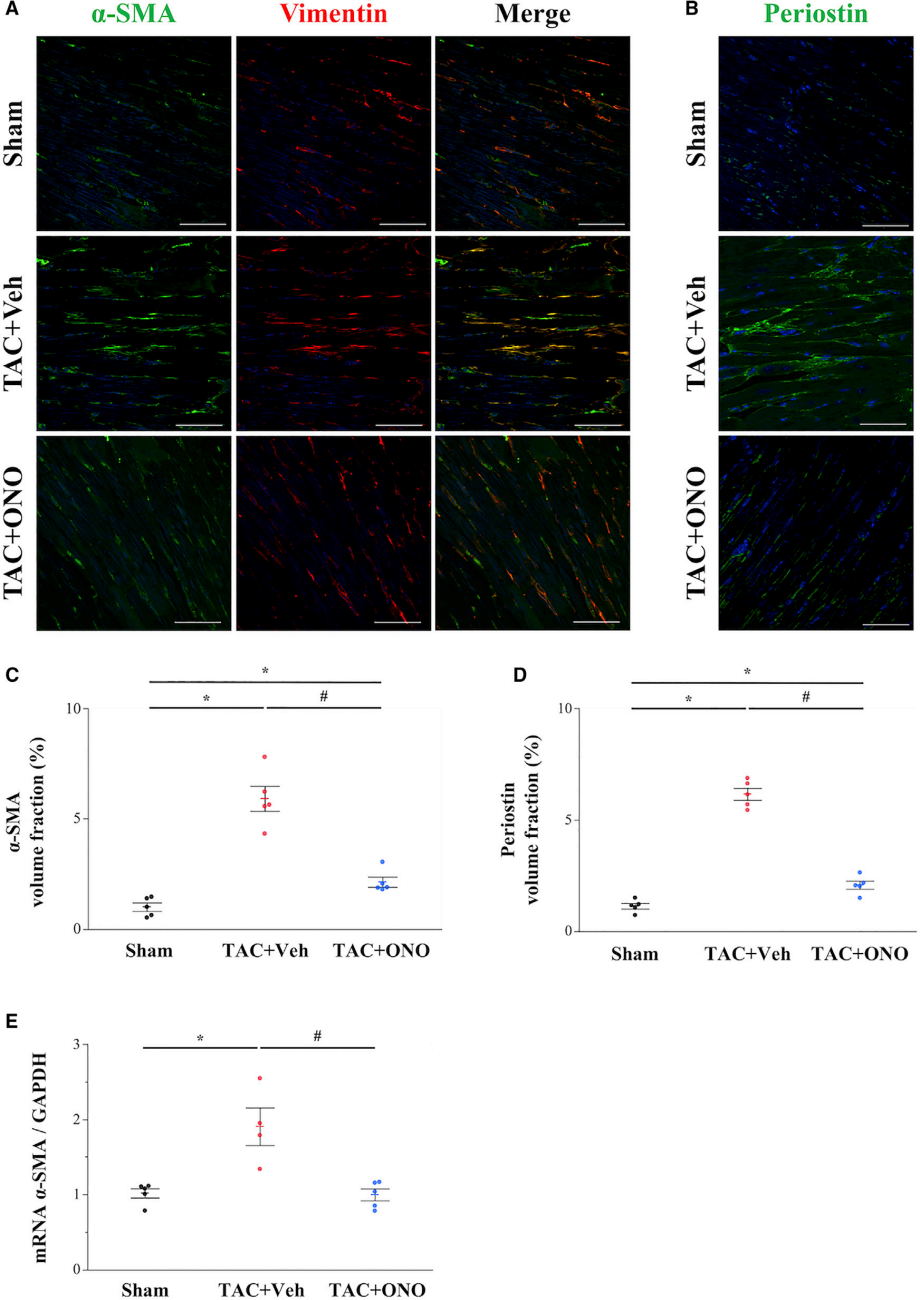
ONO-1301SR Treatment Reduces Myofibroblasts in the TAC Heart (A) Immunohistochemistry of α-smooth muscle actin (α-SMA; green) and vimentin (red). The cells positive for both α-SMA and vimentin represent cells of fibroblast origin that have transitioned into myofibroblasts. Scale bars, 50 μm. (B) Immunohistochemistry of periostin. Scale bars, 50 μm. (C) Quantification of α-SMA volume fraction (n = 5 for each group). (D) Quantification of periostin volume fraction (n = 5 for each group). (E) Gene expression level of *α-SMA* was analyzed by quantitative real-time polymerase chain reaction (PCR; n = 4‒5 for each group). Data are represented as mean ± SEM. ∗p < 0.05 versus the sham group; ^#^p < 0.05 versus the vehicle group.

Analysis of the expression of α-smooth muscle actin (α-SMA), the typical molecular marker of myofibroblasts,^21^ revealed that cells positive for both α-SMA and vimentin, which represent cells of fibroblast origin that have transitioned into myofibroblasts in the LV free wall, were significantly increased in the vehicle group, whereas ONO-1301SR treatment significantly reduced this number (Figure 4). Moreover, the α-SMA volume fraction was significantly lower in the ONO group than in the vehicle group (Figure 4C; 2.1% ± 0.2% versus 5.9% ± 0.6%, p = 0.012). Furthermore, *α-SMA* mRNA expression was significantly downregulated in the ONO compared to that in the vehicle group (Figure 4E; p = 0.020). Immunohistochemical staining for the expression of periostin (Figure 4B), a relatively new potential marker of myofibroblasts,^22^ showed that the periostin volume fraction was also significantly lower in the ONO group than in the vehicle group (Figure 4D; 2.1% ± 0.2% versus 6.2% ± 0.3%, p = 0.012). These results indicated that ONO-1301SR treatment suppresses myofibroblasts in the TAC heart.

### ONO-1301 Suppresses TGF-β-Induced FMT and Collagen Expression *In Vitro*

**Figure 5:**
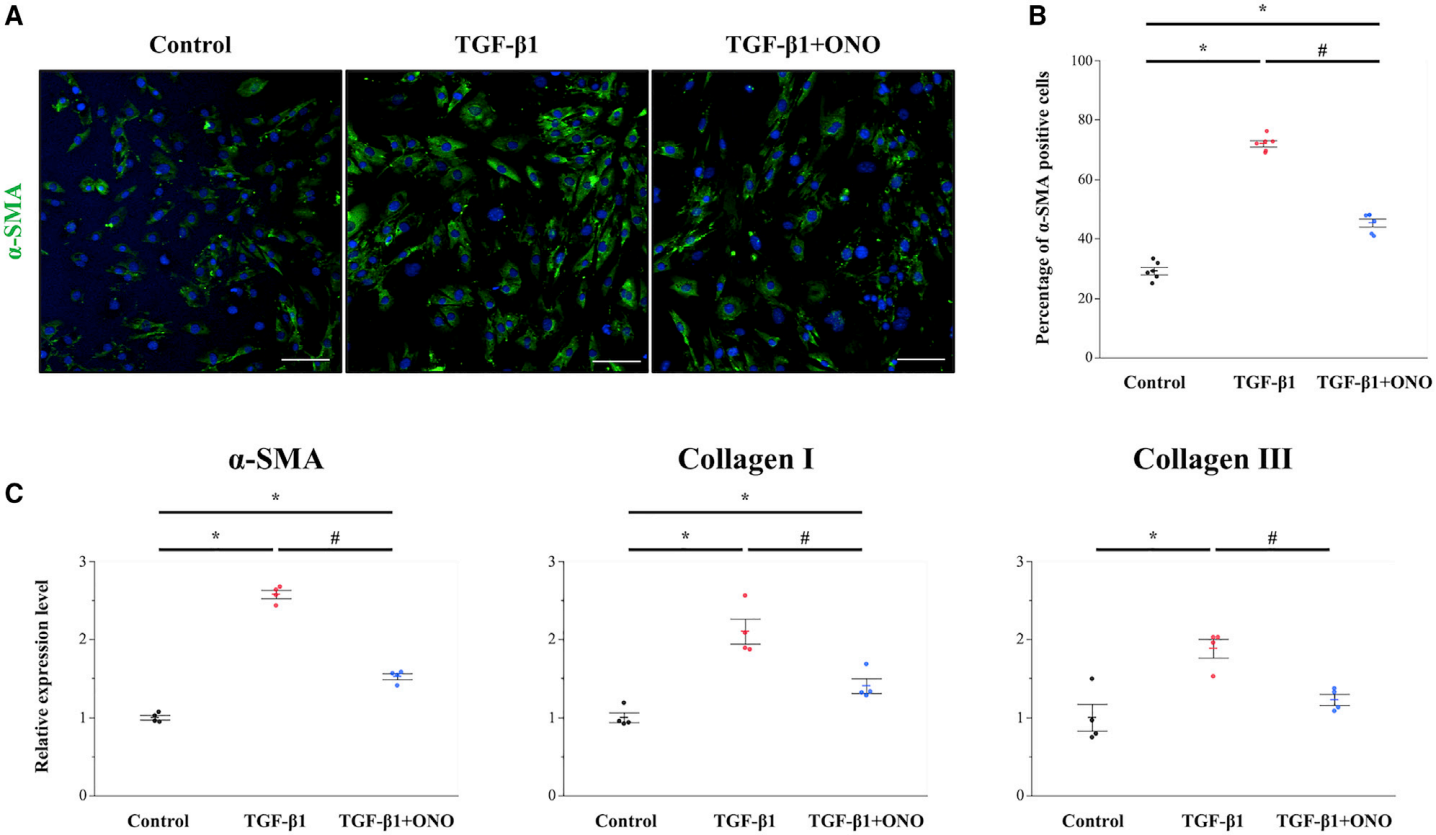
ONO-1301 Suppresses TGF-β1-Induced Fibroblast-to-Myofibroblast Transition and Collagen Expression *In Vitro* (A) Immunofluorescence staining of α-SMA (green) stimulated by TGF-β1 in the presence or absence of ONO-1301. Nuclei (blue). Scale bars, 100 μm. (B) Quantification of α-SMA-positive cells as percentage of all cells (n = 6 for each group). (C) Gene expression levels of *α-SMA*, *type I collagen*, and *type III collagen* were analyzed by quantitative real-time PCR (n = 4 for each group). Data are represented as mean ± SEM. ∗p < 0.05 versus the control group; ^#^p < 0.05 versus the TGF-β1 group.

We isolated and cultured CFs from the LV of adult mice to examine the cell type-specific effects of ONO-1301 on CFs. CFs were stimulated with TGF-β1 (10 ng/mL) in the presence or absence of ONO-1301 (100 μM) for 24 h. As a result, ONO-1301 significantly suppressed TGF-β-induced FMT as evidenced by the decreased expression of α-SMA (Figure 5A). As shown in Figure 5B, the percentage of α-SMA-positive cells was significantly increased by stimulation with TGF-β1, whereas ONO-1301 treatment significantly reduced the percentage of these cells (72.0% ± 1.1% versus 45.4% ± 1.3%, p < 0.01). In addition, the elevated gene expression levels of *α-SMA*, *Col1*, and *Col3* were significantly downregulated by ONO-1301 treatment (Figure 5C).

### ONO-1301 Inhibits TGF-β-Induced CF Proliferation and Migration *in Vitro*

**Figure 6:**
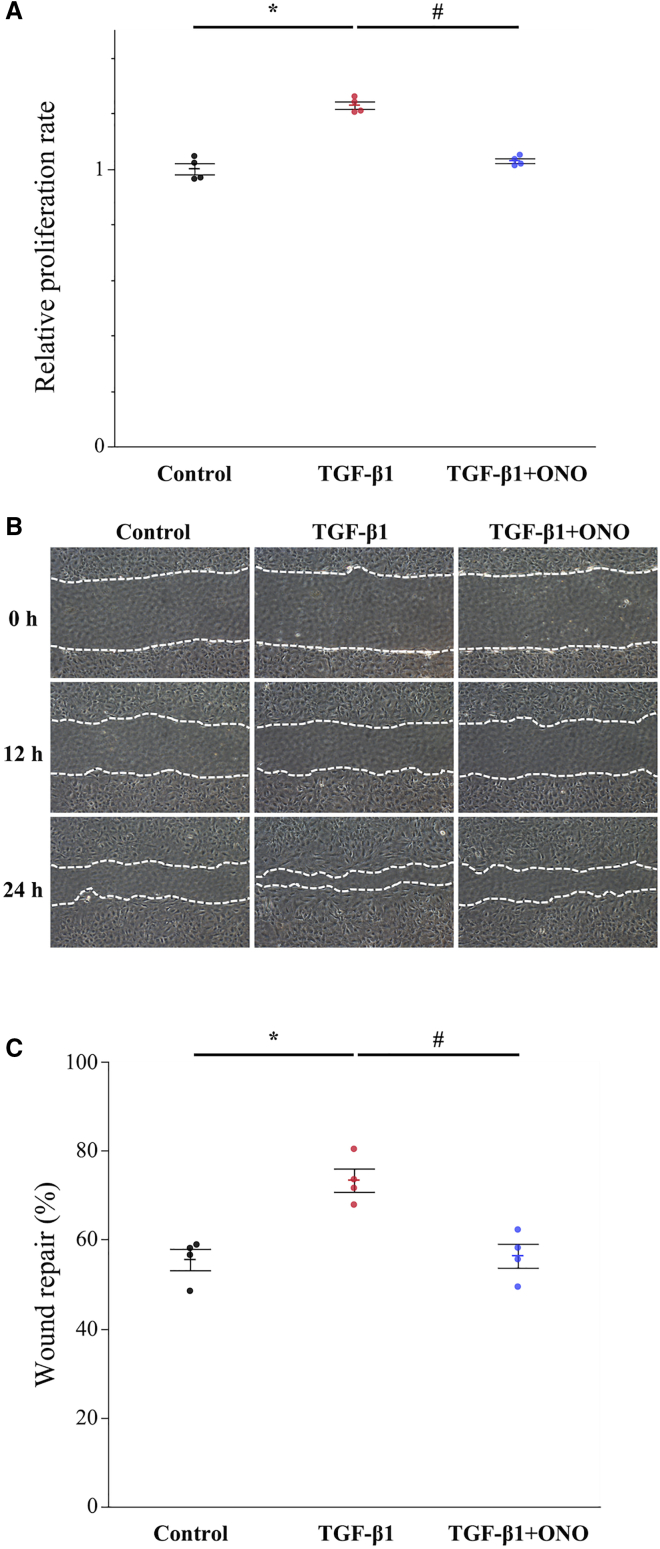
ONO-1301 Inhibits TGF-β-Induced Cardiac Fibroblast Proliferation and Migration *In Vitro* (A) The proliferation ability of cardiac fibroblasts (CFs) stimulated by TGF-β1 in the presence or absence of ONO-1301 was assessed by the Cell Counting Kit-8 (CCK-8) assay (n = 4 for each group). (B) Result of wound-healing assay; CFs were stimulated with TGF-β1 in the presence or absence of ONO-1301 and were imaged by microscopy at the indicated times. (C) Wound repair (%) was expressed as the healing area recovered by the cells. Data are represented as mean ± SEM. ∗p < 0.05 versus the control group; ^#^p < 0.05 versus the TGF-β1 group.

The proliferation ability of CFs stimulated with TGF-β1 (10 ng/mL) in the presence or absence of ONO-1301 (100 μM) was assessed using the Cell Counting Kit-8 (CCK-8; Dojindo Molecular Technologies, Kumamoto, Japan) assay. As a result, stimulation by TGF-β1 significantly accelerated CF proliferation, whereas ONO-1301 treatment significantly inhibited the TGF-β-induced CF proliferation (Figure 6A; p = 0.030). The migration ability of CFs was analyzed using a wound-healing assay. After stimulation with TGF-β1 (10 ng/mL), CFs demonstrated a strong migration capacity (Figure 6B). However, after co-culture with ONO-1301 (100 μM), the migration ability of CFs was significantly suppressed (Figure 6C; p = 0.030). These data support that ONO-1301 inhibited TGF-β-induced FMT.

## Discussion

Herein, we have demonstrated that ONO-1301SR provided an anti-fibrotic effect and attenuated pressure overload-induced systolic dysfunction *in vivo*. ONO-1301SR treatment downregulated fibrosis-related cytokines, such as Col1, Col3, TGF-β1, and CTGF, but upregulated cardioprotective cytokines, including VFGF, HGF, and SDF-1. Importantly, ONO-1301SR treatment significantly suppressed the expression levels of myofibroblast markers, including α-SMA and periostin, in the TAC heart. To clarify the cell-specific effects of ONO-1301 on CFs, we performed *in vitro* experiments, finding that ONO-1301 significantly suppressed TGF-β-induced FMT, as evidenced by the suppression of α-SMA expression and inhibition of TGF-β-induced CF proliferation and migration. These findings could support the hypothesis that ONO-1301SR attenuates cardiac fibrosis by inhibiting FMT.

ONO-1301 is a synthetic IP agonist lacking the typical prostanoid structure, which is associated with the biological and chemical stability of this agent, resulting in long-lasting prostacyclin activity.^9^ ONO-1301 also has a 3-pyridine radical, which exerts thromboxane A_2_ synthase inhibitory activity, inducing intrinsic prostaglandin I_2_ production, leading to augmentation of the IP agonistic activity. Therefore, ONO-1301 has theoretical advantages as a therapeutic drug over other synthetic prostacyclin agonists, such as epoprostenol, beraprost, iloprost, and treprostinil. Furthermore, ONO-1301SR, a PLGA-polymerized ONO-1301 was developed to achieve a further slow-releasing system. Our laboratory has reported the therapeutic effects of ONO-1301SR for various cardiac pathologies, including acute myocardial infarction (MI), chronic MI, and cardiomyopathy.^10^, ^11^, ^12^, ^13^, ^14^

Concerning the anti-fibrotic effects of ONO-1301, Nakamura et al.^23^ first reported the therapeutic effects of ONO-1301SR in a mouse model of acute MI in 2007. The anti-fibrotic effects of ONO-1301SR in a hamster model of dilated cardiomyopathy were also investigated by Hirata et al.^24^ They concluded that angiogenesis by ONO-1301-induced HGF and VEGF upregulation was the key therapeutic mechanism of ONO-1301SR treatment. Conversely, our group reported that ONO-1301SR treatment enhanced the recruitment of bone marrow-derived cells into the damaged myocardium in a mouse model of acute MI.^12^ Thus, we indicated that accumulation of bone marrow-derived cells by ONO-1301 treatment was an alternative therapeutic mechanism of this drug. In this study, we also confirmed that ONO-1301SR treatment significantly upregulated HGF, VEGF, and SDF-1 in a murine pressure-overloaded model. The induction of HGF or VEGF and the accumulation of bone marrow-derived cells were considered to be associated with the anti-fibrotic mechanism of ONO-1301 at least partly.

CFs play a central role in ECM production and the fibrotic remodeling process, and they are activated to the myofibroblast phenotype after a myocardial injury.^1^^,^^5^ Following this FMT, CFs attain increased proliferation and migration abilities and secrete elevated levels of ECM proteins.^25^ In addition, TGF-β signaling plays a key role in fibrogenesis by modulating FMT and promoting ECM accumulation.^26^ Thus, TGF-β-induced FMT is considered a key therapeutic target in cardiac fibrosis treatment; however, no study has investigated whether ONO-1301 can modulate TGF-β-induced FMT. In this study, we identified a significant reduction in the number of cells positive for both α-SMA and vimentin, which represented fibroblast-originated cells that have transitioned into myofibroblasts, in the ONO-1301SR-treated TAC hearts. Moreover, we identified that ONO-1301 co-incubation significantly suppressed TGF-β-induced FMT, as evidenced by a significant suppression of α-SMA expression and a significant inhibition of the proliferation and migration abilities of TGF-β-stimulated CFs. These findings indicated the novel anti-fibrotic effect of ONO-1301 on CFs and in inhibiting FMT, resulting in amelioration of cardiac fibrosis.

Although we demonstrated the new therapeutic effect of ONO-1301 in inhibiting FMT, the underlying mechanism is still unclear. Important issues include whether ONO-1301 directly acted on CFs to inhibit FMT or whether ONO-1301-induced HGF indirectly inhibited FMT. TGF-β reportedly binds to TGF-β receptors and activates the TGF-β/Smad signal pathway to promote FMT.^4^ Chen et al.^17^ reported that beraprost inhibits CF activation by suppressing the TGF-β/Smad signal pathway. Stratton et al.^18^^,^^27^ reported that iloprost prevents TGF-β-induced fibrotic response by inhibiting the Ras/MEK/ERK pathway. Taken together, ONO-1301 has the potential to act on CFs directly via the IP and regulate the downstream intracellular signaling of TGF-β, resulting in FMT inhibition. Conversely, HGF exerts anti-fibrotic action via its specific tyrosine kinase receptor, c-Met.^28^ Okayama et al.^29^ reported that HGF treatment inhibited TGF-β-induced FMT as indicated by suppression of α-SMA expression. Nasu et al.^16^ showed that ONO-1301 inhibited TGF-β-induced epithelial-mesenchymal transition of immortalized murine proximal tubular cells, whereas blockade of endogenous HGF diminished the effect. Thus, both the direct action of ONO-1301 on CFs and its indirect autocrine-paracrine action via HGF are considered to be involved in the mechanism of inhibiting FMT. Further investigation is necessary to reveal the precise molecular mechanisms associated with inhibition of FMT by ONO-1301.

The current study has some limitations. First, the TAC model was used to examine the specific effects on the LV; however, further investigations using other hypertensive models are necessary to translate these findings into humans. Second, the lack of dose-dependent analyses may be a limitation. We considered that 5 mg/kg was the optimum dose of ONO-1301SR based on our previous experiments.^9^^,^^10^^,^^14^ However, further investigation is needed to explore the optimal dose to obtain the maximum benefit with the minimum side effects, such as diarrhea and hypotension. Third, the observation period was relatively short. Given that our method used a single administration of ONO-1301SR, 2 weeks was considered the appropriate point to evaluate the effectiveness of ONO-1301SR; however, the long-term effects should also be evaluated. Finally, the biomechanical inputs, such as the stiffness of dishes or plates, could affect the results of the *in vitro* study. We used experimental equipment frequently used in Japan; however, information on their stiffness is not publicly available. Further investigations are necessary to optimize the environment for *in vitro* study.

In conclusion, we demonstrated that the synthetic prostacyclin agonist ONO-1301SR attenuates pressure overload-induced cardiac fibrosis. The anti-fibrotic effects of ONO-1301SR could be related to inhibiting TGF-β-induced FMT besides promoting the induction of cardioprotective cytokines. Although further research is necessary to clarify its therapeutic mechanisms in more detail, ONO-1301 can be a potential novel anti-fibrotic drug in the treatment of heart failure.

## Materials and Methods

### Animals

All experimental procedures related to animal studies were approved by the Osaka University Ethics Committee for Animal Experiments. Eight-week-old male C57BL/6J mice were purchased from CLEA Japan (Tokyo, Japan), housed in a temperature-controlled room, and were exposed to a 12-h light/12-h dark cycle, with *ad libitum* access to food and water.

### ONO-1301 and ONO-1301SR

ONO-1301SR, a slow-releasing form of ONO-1301, was generated by polymerization of ONO-1301 with PLGA as previously described.^9^ The ONO-1301 content ratio of ONO-1301SR was 16.9%, and the releasing time of ONO-1301SR was more than 14 days, as determined by the measurement of residual ONO-1301 in the pellet by liquid chromatography. As previously described, a consistently heart-dominant evaluation of ONO-1301 concentration until 4 weeks after ONO-1301SR administration *in vivo* showed that the levels of ONO-1301 in the ventricle were kept higher than those in the plasma until 2 weeks after administration.^10^

### TAC and ONO-1301SR Administration

Induction of TAC-induced pressure overload was performed as described previously.^25^ Briefly, mice were anesthetized with isoflurane (1.0%–3.0% in medical-grade oxygen). The aortic arch was exposed through a partial median sternotomy. A 5-0 silk suture was placed under the transverse aorta between the innominate artery and left common carotid artery and tied around a 27G needle. Sham-operated mice were used as the control (the sham group), and TAC-operated mice were categorized into two groups. Two weeks after TAC surgery, the mice were subcutaneously administrated 5 mg/kg of ONO-1301SR dissolved in phosphate-buffered saline (PBS) (the ONO group) or PBS only (the vehicle group). Two weeks after the administration, the echocardiographic parameters, BW, and HW were measured, and mice were sacrificed for histological and monocular analyses.

### Transthoracic Echocardiography

Transthoracic echocardiographic analyses were performed using Vivid-I (GE Healthcare, Little Chalfont, UK) under isoflurane inhalation (1%). LVDd (mm), LVDs (mm), and heart rate (beats/min) were measured. Fractional shortening (FS) was calculated as [(LVDd − LVDs)/LVDd] × 100 (%).

### Histopathological, Immunohistochemical, and Immunocytochemical Analyses

The hearts were fixed in 10% formalin and embedded in paraffin. Cardiomyocyte diameters were evaluated by periodic acid-Schiff (PAS) staining. The degree of fibrosis was determined by picrosirius red staining, and the fibrotic area was calculated as the ratio of the total interstitial fibrosis area to the total LV area of an LV section using MetaMorph software (Molecular Devices, Sunnyvale, CA, USA). Volume fractions of collagen, α-SMA, and periostin were calculated in the same manner. Optimal cutting temperature compound (Funakoshi, Tokyo, Japan)-embedded frozen heart sections and CFs were subjected to immunohistochemistry and immunocytochemistry analyses using the following antibodies: rabbit anti-Col1 (1:100; Abcam, Cambridge, UK), rabbit anti-Col3 (1:100; Abcam), mouse anti-α-SMA (1:50; Dako, Glostrup, Denmark), rabbit anti-vimentin (1:100; Abcam), and rabbit anti-periostin (1:100; Abcam). Images were obtained using confocal laser microscopy (FV1000-D IX81; Olympus, Tokyo, Japan).

### Isolation and Culture of Adult Mouse CFs

CFs were isolated from the left ventricles of 8-week-old male C57BL/6J mice by the collagenase digestion method as previously described.^26^ Briefly, the hearts were minced and digested in 1 mg/mL collagenase type II (Sigma-Aldrich, St. Louis, MO, USA) for 30 min at 37°C. Fully digested hearts were centrifuged at 30 rpm for 5 min at 4°C, and the supernatant was centrifuged at 1,000 rpm for 8 min at 4°C. The pellet was resuspended, and the cell suspension was filtered through a 70-μm cell strainer (BD Falcon, Franklin Lakes, NJ, USA). CD31-negative cells were selected using an anti-mouse CD31 antibody. CD31-negative CFs were cultured in Dulbecco’s modified Eagle’s medium (DMEM; Thermo Fisher Scientific, Waltham, MA, USA) containing 10% fetal bovine serum (FBS), 100 U/mL penicillin, and 100 μg/mL streptomycin. CD31-negative CFs were used for *in vitro* experiments after two passages and 24 h of starvation.

### Cell Proliferation Assay

CF proliferation was assessed using a CCK-8 (Dojindo Molecular Technologies, Kumamoto, Japan) assay in accordance with the manufacturer’s instructions. Briefly, CFs were seeded into 96-well plates (catalog no. 3860-096; Iwaki, Tokyo, Japan) at a density of 5,000 cells/well in 100 μL of DMEM containing 10% FBS and incubated overnight. After serum starvation for 24 h, CFs were treated with TGF-β1 (10 ng/mL; R&D Systems, Minneapolis, MN, USA) in the presence or absence of ONO-1301 (100 μM) in DMEM containing 10% FBS. After 24 h, the CCK-8 reagent was added to the culture, and CFs were incubated at 37°C for an additional 2 h. Absorbance was measured at 450 nm with a microplate reader.

### Wound-Healing Assay

CFs were seeded into six-well plates (catalog no. 3810-006; Iwaki), grown to confluence, and treated with TGF-β1 (10 ng/mL; R&D Systems) in the presence or absence of ONO-1301 (100 μM) in DMEM containing 10% FBS for 24 h. The CFs were serum starved for 4 h, and then wounds were created by scraping the cell layer using a 200-μL sterile micropipette tip. After wounding, the debris was removed by washing with PBS, and CFs were stimulated with basal medium. At 0, 12, and 24 h after injury, the wounds were visualized with an inverted microscope (IX70; Olympus) to assess cell migration ability. The experiments were repeated four times independently.

### Quantitative Real-Time PCR

Total RNA was extracted from hearts using an RNeasy fibrous tissue mini kit (QIAGEN, Venlo, the Netherlands) and from cultured cells using a PureLink RNA mini kit (Thermo Fisher Scientific) in accordance with the manufacturers’ instructions. The RNA (1 μg of each sample) was reverse-transcribed into cDNA using the SuperScript VILO cDNA synthesis kit (Thermo Fisher Scientific). Quantitative real-time PCR was performed with the ABI 7500 Fast real-time PCR system (Thermo Fisher Scientific) using TaqMan Fast advanced master mix (Thermo Fisher Scientific). The following genes were analyzed using TaqMan gene expression assays (Thermo Fisher Scientific): Col1 (catalog no. Mm00801666_g1), Col3 (catalog no. Mm01254476_m1), α-SMA (catalog no. Mm00725412_s1), IL-6 (catalog no. Mm00446190_m1), TGF-β1 (catalog no. Mm01178820_m1), CTGF (catalog no. Mm01192933_g1), MMP2 (catalog no. Mm00439498_m1), VEGF (catalog no. Mm00437306_m1), HGF (catalog no. Mm01135184_m1), and SDF-1 (catalog no. Mm00445553_m1). The expression of each mRNA was normalized to that of glyceraldehyde-3-phosphate dehydrogenase (GAPDH; catalog no. Mm99999915_g1). Relative gene expression was determined using the ΔΔCt method.

### Statistical Analysis

All statistical analyses were performed using JMP Pro 14.1.0 for MacOSX (SAS Institute, Cary, NC, USA). Continuous variables were reported as the mean and standard error of the mean. Statistical analyses were performed using nonparametric methods due to the small sample sizes. Intergroup comparisons were performed using a Kruskal-Wallis H analysis with a Mann-Whitney U test *ad hoc* analysis. p values <0.05 were considered to be statistically significant.

## Author Contributions

K.M. designed and conducted experiments and wrote the manuscript; Y.S., A.H., and T.K. supported experiments; and S.M. and Y.S. planned the research and supervised the project and the manuscript.

## Conflicts of Interest

The authors declare no competing interests.
